# Prospective longitudinal study of men who have sex with men and transgender women to determine HIV incidence in two provinces in Thailand

**DOI:** 10.1371/journal.pone.0309355

**Published:** 2024-11-14

**Authors:** Chawetsan Namwat, Warong Leela-Apiradee, Thawat Tiawilai, Nicole Dear, Tanyaporn Wansom, Suchai Kitsiripornchai, Nakorn Premsri, Siriwat Akapirat, Trevor A. Crowell, Leilani Francisco, Qun Li, Merlin L. Robb, Kirsten S. Smith, Elizabeth A. Heger, Mark M. Fukuda, Robert J. O’Connell, Supachai Rerks-Ngarm, Sandhya Vasan, for the RV348M Study Group

**Affiliations:** 1 Department of Disease Control, Ministry of Public Health, Nonthaburi, Thailand; 2 Theppharat Nakhon Ratchasima Hospital, Nakhon Ratchasima, Thailand; 3 Photharam Hospital, Ratchaburi, Thailand; 4 U.S. Military HIV Research Program, CIDR, Walter Reed Army Institute of Research, Silver Spring, MD, United States of America; 5 Henry M. Jackson Foundation for the Advancement of Military Medicine, Inc., Bethesda, Maryland, United States of America; 6 Armed Forces Research Institute of Medical Sciences, Bangkok, Thailand; 7 Walter Reed Army Institute of Research, Silver Spring, Maryland, United States of America; 8 US Army Medical Materiel Development Activity, Fort Detrick, Maryland, United States of America; HIV/STI Surveillance Research Center and WHO Collaborating Center for HIV Surveillance, Institute for Future Studies in Health, Kerman University of Medical Sciences, ISLAMIC REPUBLIC OF IRAN

## Abstract

**Background:**

In Thailand, HIV transmission is well characterized in large urban centers such as Bangkok and Chiang Mai but less so outside of these areas. The main purpose of this study was to assess HIV incidence and associated risk factors in Nakhon Ratchasima and Ratchaburi.

**Methods:**

Participants assigned male sex at birth were enrolled in this prospective observational cohort study between November 2017 and July 2018. HIV and syphilis testing and sociobehavioral questionnaires were administered over 18 months. HIV incidence rates and 95% confidence intervals (CIs) were estimated using a Poisson distribution. Cox proportional hazards models were used to estimate unadjusted and adjusted hazard ratios (aHRs) and 95% CIs for associations between potential risk factors and HIV seroconversion.

**Results:**

A total of 1003 participants were enrolled. Overall HIV incidence was 1.56 per 100 person-years (95% CI:1.02–2.44) and similar at both sites. In the fully adjusted model, sex with a sex worker in the past six months was associated with reduced risk of seroconversion (aHR:0.10, 95% CI:0.01–0.77). In the reduced adjusted model, receptive anal sex (aHR:3.40, 95% CI:1.32–8.74) and STI diagnosis in the past six months (aHR:3.58, 95% CI:1.19–10.76) were associated with seroconversion, while sex with a sex worker in the past six months was associated with reduced risk of seroconversion (aHR:0.11, 95% CI:0.02–0.67). Additionally, 56% reported interest in taking PrEP and 82% reported willingness to participate in a hypothetical future vaccine trial.

**Conclusions:**

Recent receptive anal sex practices were associated with HIV acquisition in these populations, highlighting the continued need for interventions encouraging safer anal sex practices to reduce HIV incidence.

## Introduction

The burden of HIV in Thailand is predominantly among men who have sex with men (MSM) and transgender women (TGW) [1], with much of the incidence concentrated within the capitol city of Bangkok [2, 3]. Quantifying HIV incidence is critical not only to the design of effective public health measures, but also to understanding the feasibility of longitudinal efficacy testing of interventions to prevent new HIV acquisition, including vaccines, microbicides, long-acting preventive antiretroviral therapy (ART), and immunotherapy with monoclonal antibodies.

The HIV epidemic has been well characterized in urban centers such as Bangkok and Chiang Mai [4], but less so outside of these areas. In the current study, we focused on characterizing HIV incidence and risk factors associated with HIV transmission, in two locations in Thailand where prospective HIV incidence has not previously been reported. Ratchaburi is a town west of Bangkok where HIV prevention efforts were adopted and promoted early in the epidemic [5, 6]. Nevertheless, MSM remain at high risk for HIV, due in part to commercial sex work. Nakhon Ratchasima, or Korat, is a city that is known as the gateway to the Northeast Isan region of Thailand. Although a small study estimated HIV prevalence of 9.7% among MSM in 2010 [7], there have been no subsequent incidence studies in Korat. We further surveyed opportunities for prevention interventions, including pre-exposure prophylaxis (PrEP) and potential future HIV vaccines in both locations.

The main purpose of this study was to measure HIV incidence and identify factors associated with HIV acquisition among MSM and TGW in Nakhon Ratchasima and Ratchaburi, Thailand. The secondary objectives were to describe PrEP uptake and willingness to participate in a future hypothetical vaccine trial in these key populations in order to determine whether future HIV vaccine efficacy trials might be feasibly conducted in these locations.

## Materials and methods

### Study population

Participants for this prospective observational cohort study were enrolled at two sites in Nakhon Ratchasima and Ratchaburi, Thailand. Both studies were conducted in freestanding clinic sites associated with provincial hospitals. Recruitment was conducted in partnership with local non-governmental organizations (NGOs): Boyfriend Korat in Nakhon Ratchasima and Rainbow Ratburi in Ratchaburi through peer educators who were already affiliated with these groups.

Individuals were eligible for enrollment if they were HIV-uninfected, aged 18 to 35 years old, assigned male sex at birth, resided in Nakhon Ratchasima or Ratchaburi province, and were willing to be followed for 18 months. In addition, eligible participants had to satisfy one or more of the following HIV risk criteria in the past six months: engaged in anal intercourse without a condom with a male or TGW; sexual partner known to be living with HIV or with unknown HIV status; engaged in anal intercourse with three or more male or TGW sexual partners; exchanged sex for money, gifts, shelter, or drugs; or been diagnosed with a sexually transmitted infection (STI). Exclusion criteria included previous participation in an HIV vaccine study, unless a documented placebo recipient, as well as any condition that would interfere with safe adherence to study procedures.

## Ethics approval and consent to participate

The study was approved by the institutional review boards at Walter Reed Army Institute of Research (#2376), and the Thai Ministry of Public Health Ethical Research Committee. Research was performed in accordance with the Declaration of Helsinki and appropriate Thai national guidelines. All participants gave written informed consent in Thai.

### Study procedures

Participants completed a screening visit to assess study eligibility, an enrollment visit (generally conducted on the same day as screening if the participant met eligibility criteria), and follow-up visits at three, six, twelve, and eighteen months. At all visits, personal identifying information was restricted to immediate study staff and clinical and laboratory data was de-identified prior to analysis.

At screening for study eligibility and each follow-up visit, HIV testing was conducted according to Thai National Guidelines. An initial test was conducted using Alere Determine HIV-1/2 Ag/Ab Combo (Orgenics Ltd., Yavne, Israel). Positive tests were confirmed with First Response HIV-1-2.O (Premier Medical Ltd, Daman, India) and SD Bioline HIV 1/2 3.0 (Standard Diagnostics, Inc., Gyeonggi-do, Korea). All participants received HIV risk reduction counseling, including provision of condoms and lubricant and information about PrEP, and pre-test and post-test counseling from trained study staff.

At screening for study eligibility, participants were also tested for hepatitis B surface antigen (Alere Determine HBsAg Test, Alere International Limited, Galway, Ireland), hepatitis C antibody (SD Bioline HCV, Standard Diagnostics, Inc., Gyeonggi-do, Korea), and syphilis by rapid diagnostic test (SD Bioline Syphilis 3.0, Standard Diagnostics, Inc., Gyeonggi-do, Korea). Syphilis testing was repeated at each follow-up study visit.

Standardized risk behavior and PrEP questionnaires, for HIV-uninfected participants, were administered at the screening visit and all follow-up visits using computer assisted self-interview (CASI) technology. The questionnaires captured self-reported information on sociodemographic characteristics, sexual activities, risk behaviors, and knowledge, attitudes, and usage of PrEP. Participants were asked if they would be interested in taking daily oral PrEP if it were available. Participants who indicated interest were offered PrEP through the Thai Ministry of Public Health [8]. However, PrEP access was very different in the two sites, as PrEP was available via free demonstration projects in Nakorn Ratchasima but not in Ratchaburi, where interested participants would have to travel to Bangkok to access PrEP. Those who were not interested in taking PrEP were asked reasons why and could provide multiple reasons from a predetermined set of responses.

An HIV vaccine questionnaire was administered at enrollment, to enrolled HIV-uninfected participants, to assess general knowledge about vaccines and general interest or willingness to participate in a future hypothetical HIV vaccine trial. To assess vaccine-related knowledge, participants were asked to identify the following statements as true or false: “A vaccine is used to prevent illness” and “There is an effective vaccine to prevent HIV infection.” Participants were asked reasons for willingness or unwillingness to participate in a future hypothetical HIV vaccine trial from a predetermined set of responses and could provide multiple reasons.

Participants who were diagnosed with HIV, syphilis, or hepatitis at screening were referred for appropriate medical care through the Thai national health care system.

### Outcome measures

The main outcomes of interest included HIV incidence, PrEP uptake, and willingness to participate in a future hypothetical vaccine trial. Incident HIV infections were identified through testing conducted at screening and all follow-up visits in accordance with Thai National Guidelines. Laboratory source documentation was used to complete case report forms that were then entered into the electronic data capture system. The HIV vaccine questionnaire was administered at the enrollment visit only. Responses to this questionnaire were self-reported and administered using CASI technology to reduce response bias. The main question of interest was “If a preventive AIDS vaccine trial became available for testing to determine whether it could help prevent people from acquiring HIV, would you be willing to participate in such a study to receive vaccination?” with response options of “Yes”, “No”, “Not sure” and “Refuse to answer.” PrEP uptake data were self-reported and captured at the screening visit and all follow-up visits except the enrollment visit using CASI technology. The main question of interest was “How interested would you be in taking daily oral PrEP if it were available?” with the following response options: “Very interested”, “Somewhat interested”, Somewhat uninterested” and “Not sure.”

### Statistical analyses

Wilcoxon rank-sum and Chi-squared tests were used to describe differences in sociodemographic characteristics and HIV risk factors by site (Nakhon Ratchasima or Ratchaburi). Enrollment data on vaccine knowledge and willingness to participate in a hypothetical HIV vaccine trial were analyzed descriptively. Responses from the PrEP questionnaire were also analyzed descriptively for all time points.

Visit adherence was calculated at each follow-up visit as the number of participants who completed the visit divided by the number of participants who were expected to complete the visit. The number expected to complete each visit was calculated as the number enrolled minus the number who seroconverted before that visit. HIV incidence rates and 95% confidence intervals (CIs) were estimated, using a Poisson distribution, as the number of new HIV diagnoses divided by person-years (PY) of follow-up and multiplied by 100.

Participants at risk for HIV with at least one follow-up visit after enrollment were included in time-to-event analyses. Cox proportional hazards models were used to estimate unadjusted and adjusted hazard ratios (HRs) and 95% CIs for associations between potential risk factors and seroconversion. Two adjusted models are presented. The fully adjusted model contains all potential predictors of HIV seroconversion identified *a priori* and with knowledge of the study population and setting to assess proximal indicators of risk. The reduced model was built using backwards stepwise selection with a significance level of α = 0.20 to remove variables from the fully saturated model, in order to explain the data with a minimum number of predictors; site was held in the model. The following variables were analyzed as time-varying covariates: age, income, and factors capturing behavioral and sexual risk in the 6 months prior to each visit (condom use, sex with a sex worker, receptive anal sex, transactional sex, swinging sex, alcohol use before sex, STI diagnosis). Missing responses were folded into the highest risk category for modeling.

Analyses were performed in Stata version 16.1 (StataCorp, College Station, Texas).

## Results

### Cohort characteristics

Between November 2017 and July 2018, 1065 potential participants were screened for eligibility. HIV prevalence among all potential participants screened was 4.9% and differed by site (8 (1.5%) in Ratchaburi vs 44 (8.1%) in Nakhon Ratchasima, p<0.001). HIV prevalence was higher among TGW than MSM (20 (10.9%) vs 28 (2.5%), p<0.001). The median age of those living with and without HIV at screening was similar (22.5 (interquartile range (IQR): 20–27 years vs 22 IQR: 19–26 years, p = 0.40).

Of the 1065 potential participants screened, 62 were excluded from enrollment into the study for the following reasons: HIV was diagnosed in 52 (4.9%) individuals, 6 (0.6%) had not engaged in anal intercourse with a male or TGW in the past 6 months, 1 (0.1%) resided outside of Nakhon Ratchasima or Ratchaburi province and had not engaged in anal intercourse with a male or TGW in the past 6 months, 1 (0.1%) had not engaged in condomless anal intercourse, 1 (0.1%) had an inconclusive HIV test, and 1 (0.1%) declined enrollment.

A total of 1003 participants were enrolled. Their median age was 22 years old (IQR: 19–26 years) (Table 1). Only 162 (16.2%) had completed no or some primary education, while 615 (61.3%) had completed secondary level education, 145 (14.5%) had completed vocational education, and 81 (8.1%) had completed some or more University level education. Most (n = 757; 75.5%) had never cohabitated or been married and 646 (64.4%) made 9000 Baht (approximately $300) or less per month. Among those enrolled, 45 (4.5%) tested positive for syphilis, 23 (2.3%) had hepatitis B, and 3 (0.3%) had reactive hepatitis C antibody.

**Table 1:** Participant enrollment characteristics by site.

	All (n = 1003)	Ratchaburi (n = 503)	Nakorn Ratchasima (n = 500)	p-value
**Age (years), median (IQR)**	22 (19–26)	20 (19–24)	23 (20–28)	*<0*.*001*
**Education Level**				*<0*.*001*
None/Some primary	162 (16.2%)	99 (19.7%)	63 (12.6%)	
Secondary	615 (61.3%)	284 (56.5%)	331 (66.2%)	
Vocational	145 (14.5%)	88 (17.5%)	57 (11.4%)	
Some/Completed university/More	81 (8.1%)	32 (6.4%)	49 (9.8%)	
**Marital Status**				*0*.*097*
Never married or cohabited	757 (75.5%)	365 (72.6%)	392 (78.4%)	
Married	26 (2.6%)	16 (3.2%)	10 (2.0%)	
Cohabitating	133 (13.3%)	78 (15.5%)	55 (11.0%)	
Separated, divorced, widowed	87 (8.7%)	44 (8.7%)	43 (8.6%)	
**Monthly Income**				*<0*.*001*
≤9,000 Baht	646 (64.4%)	348 (69.2%)	298 (59.6%)	
>9,000 Baht	306 (30.5%)	114 (22.7%)	192 (38.4%)	
Missing	51 (5.1%)	41 (8.2%)	10 (2.0%)	
**Gender identity**				*<0*.*001*
Cisgender MSM	777 (77.5%)	384 (76.3%)	393 (78.6%)	
Transgender MSM	162 (16.2%)	64 (12.7%)	98 (19.6%)	
Unknown/Missing	64 (6.4%)	55 (10.9%)	9 (1.8%)	
**Ever had forced sex**				*0*.*004*
Yes	142 (14.2%)	55 (10.9%)	87 (17.4%)	
No	838 (83.5%)	433 (86.1%)	405 (81.0%)	
Missing	23 (2.3%)	15 (3.0%)	8 (1.6%)	
**Used a condom at last sex**				*0*.*56*
Yes	388 (38.7%)	187 (37.2%)	201 (40.2%)	
No	579 (57.7%)	290 (57.7%)	289 (57.8%)	
Missing	36 (3.6%)	26 (5.2%)	10 (2.0%)	
**Had sex with a sex worker in past 6 months**				*<0*.*001*
Yes	341 (34.0%)	198 (39.4%)	143 (28.6%)	
No	625 (62.3%)	278 (55.3%)	347 (69.4%)	
Missing	37 (3.7%)	27 (5.4%)	10 (2.0%)	
**Had receptive anal sex in past 6 months**				*<0*.*001*
Yes	217 (21.6%)	79 (15.7%)	138 (27.6%)	
No	774 (77.2%)	417 (82.9%)	357 (71.4%)	
Missing	12 (1.2%)	7 (1.4%)	5 (1.0%)	
**Engaged in transactional sex in past 6 months**				*0*.*013*
Yes	408 (40.7%)	220 (43.7%)	188 (37.6%)	
No	556 (55.4%)	255 (50.7%)	301 (60.2%)	
Missing	39 (3.9%)	28 (5.6%)	11 (2.2%)	
**Had swinging sex in past 6 months**				*0*.*014*
Yes	86 (8.6%)	31 (6.2%)	55 (11.0%)	
No	862 (85.9%)	430 (85.5%)	432 (86.4%)	
Missing	55 (5.5%)	42 (8.3%)	13 (2.6%)	
**In the past 6 months, had alcoholic drinks prior to anal/neovaginal sex**				*<0*.*001*
Never drank alcohol	241 (24.0%)	128 (25.4%)	113 (22.6%)	
Did not have alcohol before sex	201 (20.0%)	143 (28.4%)	58 (11.6%)	
Had alcohol before sex	554 (55.2%)	227 (45.1%)	327 (65.4%)	
Missing	7 (0.7%)	5 (1.0%)	2 (0.4%)	
**Been diagnosed with an STI in past 6 months**				*0*.*33*
Yes	33 (3.3%)	14 (2.8%)	19 (3.8%)	
No	936 (93.3%)	477 (94.8%)	459 (91.8%)	
Missing	34 (3.4%)	12 (2.4%)	22 (4.4%)	
**Ever been tested for HIV**				*0*.*004*
Yes	369 (36.8%)	205 (40.8%)	164 (32.8%)	
No	617 (61.5%)	284 (56.5%)	333 (66.6%)	
Missing	17 (1.7%)	14 (2.8%)	3 (0.6%)	

All data are presented as n (column percentage) unless otherwise specified. P-values were calculated using Wilcoxon rank-sum or Chi-square tests. Responses labeled ‘missing’ also include ‘don’t know’ and ‘refuse to answer’ responses.

### HIV Incidence and study retention

**Fig 1:**
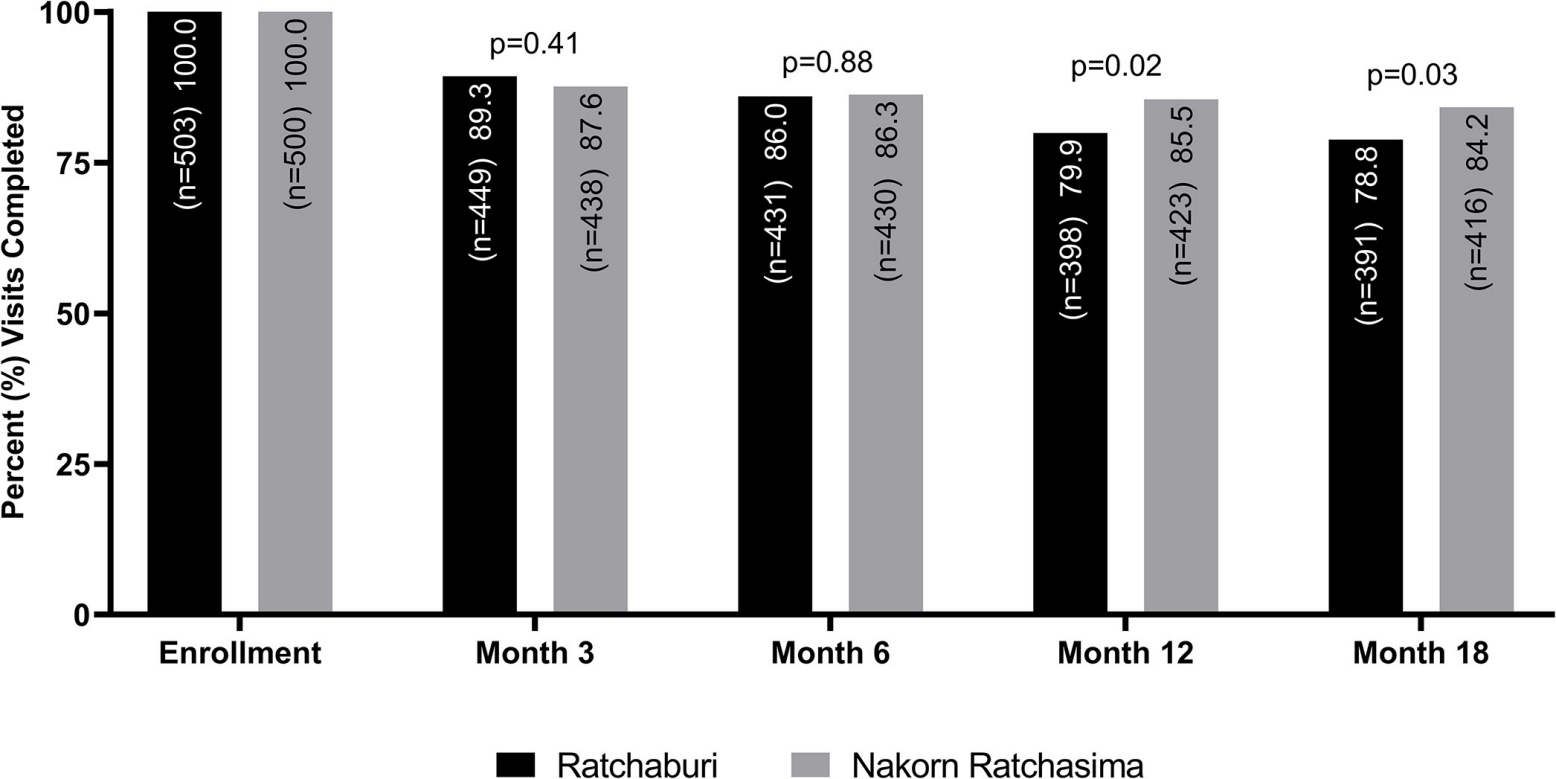
Visit adherence by site. Visit adherence was calculated at each follow-up visit as the number of participants who completed the visit divided by the number of participants who were expected to complete the visit. The number expected to complete each visit was calculated as the number enrolled minus the number who seroconverted before that visit. P-values were calculated using proportion tests for each visit.

Over the 18-month study period, 20 incident HIV infections were identified. Overall, HIV incidence was 1.56 per 100 PY (95% CI: 1.02–2.44). HIV incidence was similar at both sites, with an incidence in Ratchaburi of 1.58 per 100 PY (95% CI: 0.85–2.94) and in Nakorn Ratchasima of 1.57 per 100 PY (95% CI: 0.84–2.91) (log-rank test, p = 0.59). Overall visit adherence at 18 months was 81.5% and was higher in Nakorn Ratchasima (84.2%) than in Ratchaburi (78.8%; p = 0.03) (Fig 1).

### Factors associated with HIV seroconversion

In the unadjusted analysis, having ever previously been tested for HIV was associated with an increased risk of HIV seroconversion compared to those who had never been tested for HIV (HR: 2.47, 95% CI: 1.01–6.04) (model 1, Table 2). Increased risk of HIV seroconversion was also observed among participants who had receptive anal sex in the past six months (HR: 3.04, 95% CI: 1.26–7.29) and had been diagnosed with an STI in the past six months (HR: 5.05, 95% CI: 1.83–13.95).

**Table 2:** Unadjusted and adjusted hazard ratios of risk factors for HIV seroconversion.

	Model 1: Unadjusted HR (95% CI)	Model 2: Adjusted HR (95% CI)	Model 3: Adjusted HR (95% CI)
**Site**			
Ratchaburi	Ref	Ref	Ref
Nakorn Ratchasima	1.28 (0.52–3.17)	1.16 (0.42–3.22)	1.01 (0.38–2.65)
**Education Level**			
None/Some primary	Ref	Ref	
Secondary	1.67 (0.38–7.41)	1.79 (0.39–8.32)	
Vocational	1.46 (0.24–8.76)	1.37 (0.22–8.69)	
Some/Completed university/More	1.74 (0.25–12.36)	1.04 (0.13–8.41)	
**Marital Status**			
Never married or cohabited	Ref	Ref	
Ever married or cohabited	1.60 (0.64–4.02)	1.63 (0.59–4.46)	
**Gender identity**			
Cisgender MSM	Ref	Ref	
Transgender MSM/Unknown	2.19 (0.89–5.35)	0.91 (0.30–2.79)	
**Ever had forced sex**			
Yes/Unknown	1.73 (0.63–4.75)	0.92 (0.29–2.91)	
No	Ref	Ref	
**Ever been tested for HIV**			
Yes	**2.47 (1.01–6.04)**	1.61 (0.60–4.29)	
No/Unknown	Ref	Ref	
**Age (years)**	1.03 (0.94–1.13)	1.03 (0.93–1.15)	1.04 (0.94–1.15)
**Monthly Income**			
≤9,000 Baht/Unknown	Ref	Ref	Ref
>9,000 Baht	0.74 (0.30–1.87)	0.54 (0.19–1.51)	0.51 (0.18–1.40)
**Used a condom at last sex**			
Yes	1.48 (0.59–3.70)	1.24 (0.45–3.44)	
No/Unknown	Ref	Ref	
**Had sex with a sex worker in past 6 months**			
Yes/Unknown	0.39 (0.09–1.67)	**0.10 (0.01–0.77)**	**0.11 (0.02–0.67)**
No	Ref	Ref	Ref
**Had receptive anal sex in past 6 months**			
Yes/Unknown	**3.04 (1.26–7.29)**	2.97 (0.96–9.12)	**3.40 (1.32–8.74)**
No	Ref	Ref	Ref
**Engaged in transactional sex in past 6 months**			
Yes/Unknown	1.62 (0.62–4.21)	1.84 (0.52–6.46)	
No	Ref	Ref	
**Had swinging sex in past 6 months**			
Yes/Unknown	1.96 (0.65–5.89)	3.02 (0.69–13.15)	3.77 (0.97–14.65)
No	Ref	Ref	Ref
**In the past 6 months, had alcoholic drinks prior to anal/neovaginal sex**			
Never drank alcohol	Ref	Ref	
Did not have alcohol before sex	0.42 (0.14–1.26)	0.60 (0.19–1.88)	
Had alcohol before sex/Unknown	0.79 (0.28–2.22)	0.86 (0.29–2.54)	
**Been diagnosed with an STI in past 6 months**			
Yes/Unknown	**5.05 (1.83–13.95)**	2.94 (0.93–9.32)	**3.58 (1.19–10.76)**
No	Ref	Ref	Ref

Model 1: Unadjusted Cox proportional hazards models; Model 2: Fully adjusted Cox proportional hazards model; Model 3: Reduced adjusted Cox proportional hazards model. Participants at risk for HIV with at least one follow-up visit after enrollment were included in time-to-event analyses. Cox proportional hazards models were used to estimate unadjusted and adjusted hazard ratios (HRs) and 95% CIs for associations between potential risk factors and seroconversion. Two adjusted models are presented. The fully adjusted model contains all potential predictors of HIV seroconversion identified *a priori* and with knowledge of the study population and setting to assess proximal indicators of risk. The reduced model was built using backwards stepwise selection with a significance level of α = 0.20 to remove variables from the fully saturated model, in order to explain the data with a minimum number of predictors; site was held in the model. The following variables were analyzed as time-varying covariates: age, income, and factors capturing behavioral and sexual risk in the 6 months prior to each visit (condom use, sex with a sex worker, receptive anal sex, transactional sex, swinging sex, alcohol use before sex, STI diagnosis). Bold indicates significance at p<0.05.

In the fully adjusted model, sex with a sex worker in the past six months was significantly associated with a reduced risk of HIV seroconversion (aHR: 0.10, 95% CI: 0.01–0.77), although this was not significant in the unadjusted model (model 2, Table 2). Having ever been tested for HIV (aHR: 1.61, 95% CI: 0.60–4.29), receptive anal sex in the past 6 months (aHR: 2.97, 95% CI: 0.96–9.12), and STI diagnosis in the past 6 months (aHR 2.94, 95% CI: 0.93–9.32) lost significance in the fully adjusted model.

**Fig 2:**
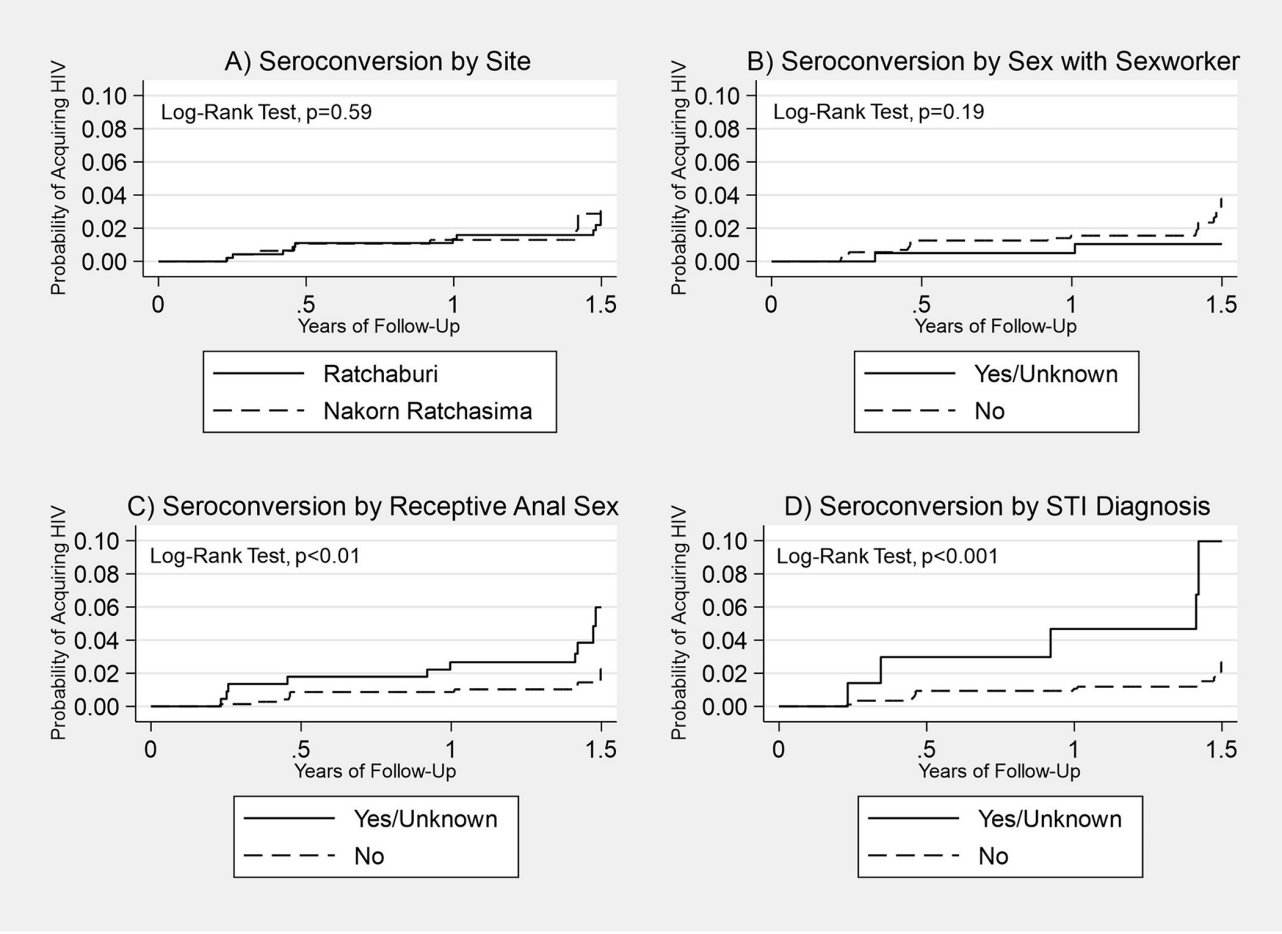
Kaplan Meier curves for probability of acquiring HIV. Probability of Acquiring HIV by (A) Site, (B) Sex with a sex worker in the past six months, (C) Engaged in receptive anal sex in past six months, and (D) Been diagnosed with an STI in past 6 months.

In the reduced model, adjusting for site, age, income, sex with a sex worker, receptive anal sex, swinging sex, and an STI diagnosis, receptive anal sex (aHR: 3.40, 95% CI: 1.32–8.74) and STI diagnosis in the past six months (aHR: 3.58, 95% CI: 1.19–10.76) were significantly associated with an increased risk of HIV seroconversion (model 3, Table 2). Similar to the fully adjusted model, sex with a sex worker in the past six months was significantly associated with a reduced risk of HIV seroconversion after adjustment (aHR: 0.11, 95% CI: 0.02–0.67). Kaplan Meier curves are depicted in Fig 2.

### Vaccine knowledge and willingness to participate

At enrollment, 806 (80.4%) participants responded correctly to the following true or false question: “A vaccine is used to prevent illness.” Only 331 (33.0%) had ever received education or information about HIV vaccine research, and 822 (82.0%) thought there was an effective vaccine to prevent HIV. However, 826 (82.4%) stated they would be willing to participate in a future hypothetical HIV vaccine trial, with 140 (14.0%) reporting they did not know if they would be willing to participate, 14 (1.4%) reporting they would not be willing to participate, and 23 (2.3%) did not respond or refused to answer.

**Fig 3:**
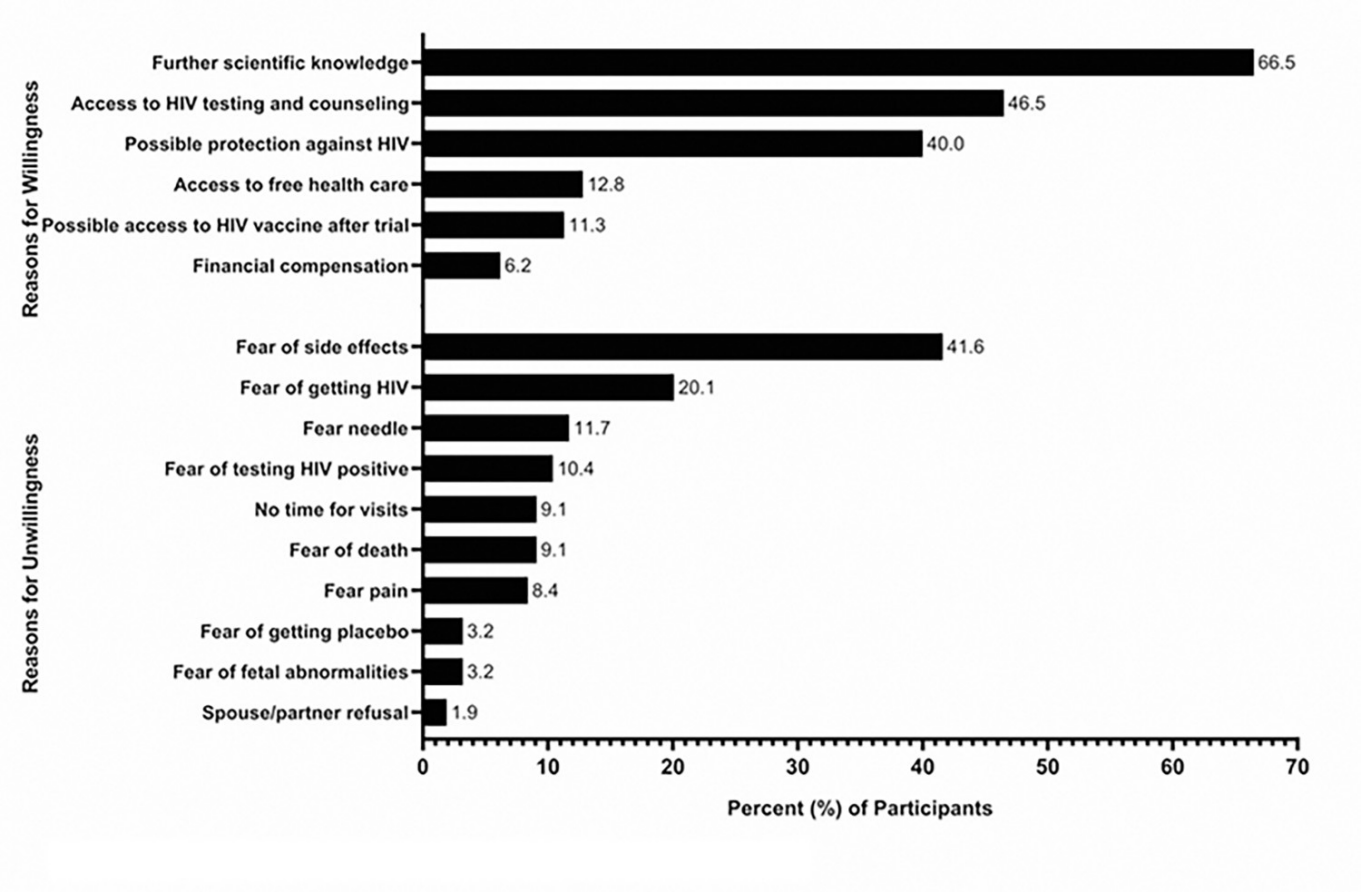
Reasons for willingness or unwillingness to participate in a vaccine trial. Participants were asked reasons for willingness or unwillingness to participate in an HIV vaccine trial from a predetermined set of responses; participants could select multiple reasons.

Among those reporting they would not be willing or were unsure if they would be willing to participate in a hypothetical HIV vaccine trial, the top three reasons for unwillingness or uncertainty included: fear of side effects (n = 64, 41.6%), fear of getting HIV (n = 31, 20.1%), and fear of needles (n = 18, 11.7%) (Fig 2). Among those reporting they would be willing to participate in a hypothetical HIV vaccine trial, the top three reasons for willingness included: to further scientific knowledge (n = 549, 66.5%), access to HIV testing and counseling (n = 384, 46.5%), and possible protection against HIV (n = 330, 40.0%) (Fig 3).

### PrEP knowledge, attitudes and usage

At screening, 97 (9.7%) participants had ever received information about PrEP and six (0.6%) had ever taken PrEP, four of whom had taken PrEP in the past month. An additional four participants from Nakorn Ratchasima initiated daily oral PrEP during the study. Of these, one stopped after one month and the other three continued throughout the duration of the study. All participants received risk reduction counseling at screening and follow-up visits, which included information about PrEP. At screening, 558 (55.6%) participants reported being very or somewhat interested in taking daily oral PrEP if it were available. Interest remained stable over the course of follow-up with 516 (58.2%) at the 3-month visit, 505 (58.7%) at the 6-month visit, 466 (56.8%) at the 12-month visit and 467 (57.9%) at the 18-month visit stating they would be very or somewhat interested in taking PrEP.

At screening, 87 (8.7%) participants reported being uninterested in taking PrEP even if it was available; 84 (9.5%) at the 3-month visit, 70 (8.1%) at the 6-month visit, 79 (9.6%) at the 12-month visit, and 74 (9.2%) at the 18-month visit, reported being uninterested in taking PrEP. Among the 229 unique participants reporting being uninterested in taking PrEP, reasons for unwillingness across all visits included: don’t want to take medicine (n = 94, 41.1%), don’t think that I am at risk for getting infected with HIV (n = 62, 27.1%), concern about side effects of taking medicine everyday (n = 49, 21.4%), cost of medicine (n = 33, 14.4%), concern about what other people will think (n = 15, 6.6%), don’t want to take medicine that contains anti-HIV medicine (n = 14, 6.1%), and don’t think that PrEP is effective (n = 5, 2.2%). Participants could provide more than one reason for being unwilling to take PrEP and 62 (27.1%) did not know or did not provide a reason for why they were uninterested in taking PrEP.

## Discussion

Thailand has been a global leader in the response to the HIV epidemic in behavioral prevention, microbicides, and in the conduct of pivotal efficacy trials of preventive vaccine candidates [9–11]. However, the bulk of these studies were conducted based on incidence information gathered in larger urban centers such as Bangkok, Pattaya, and Chiang Mai, where the epidemic is concentrated. Updated information on HIV incidence is necessary to monitor and respond to the HIV epidemic both within and outside of large urban centers. This study contributes HIV incidence information for two provinces outside of Bangkok, Ratchaburi and Nakorn Ratchasima. These sites were chosen based on activities around commercial sex work and provincial hospitals having an established history in working with community-based organizations serving key populations at risk for HIV. These community-based organizations played a key role in recruitment and retention of participants.

HIV incidence was similar at both sites, with an incidence rate in Ratchaburi of 1.58 per 100 PY and in Nakorn Ratchasima of 1.57 per 100 PY, both lower than previously reported among young MSM in Bangkok (7.4 per 100 PY) [2, 4, 12, 13]. While these studies have identified young MSM at highest risk for HIV, we did not observe any statistically significant differences in HIV seroconversion by age. However, this cohort was very young overall, with a median age of 22 years.

Although PrEP knowledge and usage was very low at the beginning of the study, over half said they would be interested in taking PrEP. However, only four participants initiated PrEP during the study despite one site having PrEP demonstration projects available. Thus, barriers to access were not the sole factor in choosing not to initiate PrEP. Similarly, less than a third of participants had ever received information about vaccine research; however, 82% reported being willing to participate in a future hypothetical HIV vaccine trial. This finding is promising and could indicate high levels of PrEP uptake should it become widely accessible and vaccine uptake should an HIV vaccine become widely available. This study could be useful as a baseline HIV incidence assessment that will benchmark falling incidence rates as PrEP is implemented more widely in these regions. Consistent with prior studies, low self-perceived risk was a key barrier to PrEP interest and will need to be addressed to optimize deployment of PrEP or an effective HIV vaccine [14]. Concerns about taking medication and experiencing side-effects, were also identified as reasons for choosing not to initiate PrEP and could be a focus of enhanced education and counseling to reduce barriers to PrEP uptake. Taken together, it is clear that focused and early prevention efforts and engagement with young MSM and TGW remain critical to curbing HIV transmission in these populations, and that these efforts can be further expanded outside of the major urban centers.

Consistent with other literature, receptive anal intercourse was significantly and independently associated with incident HIV-infection. The risk of HIV transmission via anal intercourse has been well described, though may be modified by frequency of sex acts, ART use and viral load of a partner living with HIV [15]. It should be noted that this study enrolled only individuals engaging in anal sex; however, although anal sex of any type was a criterion for inclusion, those engaging in receptive anal sex were found to be at increased risk for HIV seroconversion as compared to those not engaging in receptive anal sex. Recent STI diagnosis was associated with HIV seroconversion and may indicate shared behavioral factors that contribute to HIV and other STI acquisition. Evidence from studies conducted in other settings suggests that STIs, particularly rectal STIs, may increase the risk of HIV acquisition through biologically mediated pathways [16–18]. This finding provides support for screening and treatment of STIs as part of a comprehensive package for HIV prevention.

Sex with a sex worker was found to be associated with a lower risk of HIV seroconversion after adjustment. Other studies have observed high levels of consistent condom use among female sex workers, particularly with nonregular partners [19]. Additionally, a pooled analysis of Demographic and Health Survey data from 29 sub-Saharan African countries found that consistent condom use among men paying for sex was overall high, 84.0% (CI: 80.4–87.6), but varied by demographic characteristics [20]. High levels of condom use with sex workers may help to partially explain this unanticipated finding, despite less than 40% reporting condom use at last sex with any partner at enrollment. However, we were unable to evaluate risk differences by sex worker gender or type of sex (receptive vs insertive), which might be important in this population with already high engagement in HIV risk behaviors. Additional research is needed to further explore this association in this setting.

Although we did not find a significant relationship between condom use at last sex and HIV seroconversion, this may be due to several factors. Condom use at the last prior sexual exposure may not be an accurate representation of risk, especially in those individuals with frequent sexual risk behavior. Given that behavioral data was based on self-report, data could potentially be influenced by social desirability or recall bias. Additionally, robust associations may have been difficult to detect due to relatively small number of HIV seroconversion events. Furthermore, stepwise regression may exacerbate collinearity, fail to identify explanatory variables that have causal effects, and identify spurious associations. Finally, individuals enrolled and retained in a longitudinal cohort may be more motivated to engage in health promoting and HIV prevention activities and less inclined to engage in risky behaviors. This, in addition to narrower enrollment criteria on younger age, may potentially limiti the generalizability of our finding to other populations of MSM and TGW. Furthermore, due to the small number of HIV seroconversion events, it may be that too many potential predictors were included in the fully adjusted model to detect significant associations.

## Conclusions

This prospective incidence study in two provinces quantifies the spread of HIV in populations well outside of the capital region of Bangkok. The relatively low knowledge of PrEP warrants further outreach and education to these key populations and communities. Coupling HIV counseling and education with other STI services may be of benefit. Low PreP uptake and high willingness to participate in future vaccine studies may indicate that these locations are worthy of consideration for future efficacy trials, although the HIV incidence in this study is notable lower than reported in major urban centers such as Bangkok and Chiang Mai.

## Abbreviations

ART: antiretroviral therapy
CASI: computer assisted self-interview
CIs: confidence intervals
HRs: hazard ratios
MSM: men who have sex with men
PrEP: pre-exposure prophylaxis
PY: person-years
STI: sexually transmitted infection
TGW: transgender women
